# Long non-coding RNA NEAT1 mediated lupus nephritis induced podocytes pyroptosis through DNMT1–STING axis

**DOI:** 10.1080/0886022X.2025.2595372

**Published:** 2025-12-28

**Authors:** Yi Zeng, Zhen-Kun He, Yun-Juan Liao, Ang Ma, Cheng Li, Ping Fu

**Affiliations:** a Department of Nephrology, The Second Affiliated Hospital of Kunming Medical University, Yunnan, China; b Department of Rheumatology and Immunology, The Second Affiliated Hospital of Kunming Medical University, Yunnan, China

**Keywords:** Lupus nephritis, podocyte, NEAT1, STING, DNMT1, pyroptosis

## Abstract

Lupus nephritis (LN), a severe complication of systemic lupus erythematosus, is characterized by podocyte injury mediated through autoantibody deposition, yet the underlying mechanisms remain incompletely understood. This study investigated the role of long non-coding RNA nuclear enriched abundant transcript 1 (NEAT1) in podocyte pyroptosis and its interaction with the stimulator of interferon genes (STING) pathway. Using peripheral blood mononuclear cells (PBMCs) and IgG isolated from 25 LN patients and 20 healthy controls, we found NEAT1 expression was significantly elevated in LN patient PBMCs and confirmed this in MRL/lpr mice. *In vitro* experiments demonstrated that LN-IgG treatment induced dose-dependent podocyte pyroptosis in human podocytes, an effect enhanced by NEAT1 overexpression and suppressed by its knockdown. Further analysis revealed STING expression correlated with NEAT1 levels, and STING knockdown attenuated pyroptosis. Mechanistically, we identified that NEAT1 bound directly to dysregulation of DNA methyltransferase 1 (DNMT1), with RNA immunoprecipitation (RIP)-qPCR showing a 4.3-fold enrichment (*p* < 0.001), and observed significant DNMT1 upregulation in LN. Functional studies demonstrated that DNMT1 overexpression counteracted NEAT1’s regulatory effect on STING, establishing the NEAT1/DNMT1/STING signaling axis as a crucial regulator of podocyte pyroptosis in LN. These findings identify this novel signaling axis as a promising therapeutic target for antibody-mediated glomerular injury.

## Introduction

Systemic lupus erythematosus (SLE) is a chronic autoimmune disease characterized by systemic inflammation and multi-organ damage, with lupus nephritis (LN) representing one of its most severe complications [1]. Nearly, half of SLE patients develop LN, marked by immune complex deposition in the glomeruli, leading to podocyte injury, proteinuria, and progressive renal dysfunction [2–5]. Podocytes, terminally differentiated glomerular epithelial cells, are critical for maintaining the integrity of the filtration barrier [6,7]. Emerging evidence highlights pyroptosis – a lytic, inflammatory form of programmed cell death mediated by gasdermin D (GSDMD) – as a key driver of podocyte loss in LN. This process involves NLRP3 inflammasome activation and subsequent release of pro-inflammatory cytokines such as IL-1β and IL-18, perpetuating renal damage [8–10]. Despite advances in immunosuppressive therapies, the molecular mechanisms linking autoantibodies to podocyte pyroptosis remain poorly defined, underscoring the need for novel therapeutic targets.

Recent studies have implicated the stimulator of interferon genes (STING) pathway in autoimmune pathologies. STING, a cytosolic DNA sensor, is aberrantly activated in SLE, driving type I interferon production and amplifying inflammatory responses [11,12]. In lupus-prone mice, STING hyperactivity exacerbates glomerular injury, suggesting its central role in LN progression [13]. Concurrently, dysregulation of DNA methyltransferase 1 (DNMT1) – an enzyme critical for maintaining CpG methylation – has been observed in SLE patients, correlating with global hypomethylation and autoimmune gene activation [14]. Intriguingly, long non-coding RNAs (lncRNAs), such as nuclear enriched abundant transcript 1 (NEAT1), are increasingly recognized as epigenetic regulators in autoimmune diseases [15,16]. NEAT1 promotes NLRP3 inflammasome activation in diabetic nephropathy and enhances macrophage inflammation in SLE [17–19]. However, its role in LN-associated podocyte pyroptosis and potential interaction with STING or DNMT1 remain unexplored.

In this study, we identify NEAT1 as a key mediator of LN-IgG-induced podocyte pyroptosis through STING upregulation. We demonstrate that NEAT1 recruits DNMT1 to epigenetically potentiate STING expression, establishing a novel regulatory axis in antibody-mediated glomerular injury. These findings not only elucidate the mechanistic link between autoantibodies and podocyte death but also highlight the therapeutic potential of targeting the NEAT1/DNMT1/STING pathway in LN.

## Materials and methods

### Clinical sample collection

Peripheral blood samples were collected from 25 treatment-naïve LN patients (newly diagnosed by SLEDAI-2000 criteria: activity score >15, 24-h proteinuria > 0.5 g) and 20 healthy donors at the Second Affiliated Hospital of Kunming Medical University (January–December 2023). Detailed clinical characteristics are provided in Supplementary material. Blood collection was conducted under ethical approval (IRB No. PJ-2023-83) with written informed consent. Plasma was isolated using K2-EDTA tubes (BD Biosciences, Franklin Lakes, NJ) and stored at −80 °C for subsequent analysis of NEAT1, STING, DNMT1, IL-1β, IL-18 expression, and IgG purification.

### IgG purification and quantification

LN patient IgG was isolated via protein G affinity chromatography (AbPur rProtein G Beads Kit, Smart-Life Science, Changzhou, China) following manufacturer protocols. The concentration of purified IgG was measured with Bio-Rad Bradford Assay (Hercules, CA) by following manufacturer’s instructions.

### PBMC isolation

Whole blood (8–10 mL per subject) was collected from LN patients and healthy donors into K2-EDTA anticoagulant tubes. Samples were processed within 2 h of collection to maintain cell viability. Blood diluted 1:1 with phosphate-buffered saline (PBS) containing 2% fetal bovine serum (FBS, Gibco, Waltham, MA). Carefully layered over Ficoll-Paque PLUS (GE Healthcare, #17-1440-02, Chicago, IL) at a 3:2 ratio (diluted blood: Ficoll). Centrifuged at 400 × *g* for 30 min at 20 °C with brake disengaged. The opaque interface layer (mononuclear cells) transferred to a new 15 mL conical tube. Cells washed twice with PBS/2% FBS (centrifugation: 300 × *g*, 10 min, 4 °C). Erythrocyte lysis performed using ACK Lysing Buffer (Gibco, Waltham, MA, #A10492-01) for 2 min at RT (if residual RBCs observed). Peripheral blood mononuclear cells (PBMCs) resuspended in TRIzol LS Reagent (Invitrogen, #10296028, Waltham, MA) for RNA extraction. Viability assessed by Trypan blue exclusion (>95% required).

### Cell culture and treatment

Human podocytes (HPCs, gift from an anonymous collaborator) were maintained in RPMI-1640 (Biological Industries, Kibbutz Beit Haemek, Israel) supplemented with 10% FBS and insulin–transferrin–selenium (ITS) at 33 °C/5% CO_2_. Differentiation was induced by ITS removal and temperature shift to 37 °C for 10 days. Differentiated podocytes were treated with LN-IgG (100–1,500 μg/mL). The NEAT1 and DNMT1 overexpression plasmid was constructed using the pcDNA3.0 skeleton, and the NEAT1 sequence was obtained from NCBI. Lentiviral constructs (sh-NEAT1: LV70082351; sh-STING: LV22122202; Hanbio Technology, Shanghai, China) were transfected using polybrene (8 μg/mL). Human-NEAT1 target sequence: GCTGAGGCAGAAGAATCACTT. Human-STING target sequence: GTTTACAGCAACAGCATCTAT.

### Animal studies

All animal studies were approved by Animal Ethics Committee of The Second Affiliated Hospital of Kunming Medical University (No. kyfey2023005). Female MRL/MpJ and MRL/lpr mice (8 weeks, 18–21 g, SPF Biotechnology, Beijing, China) were housed under specific pathogen-free conditions (12 h light/dark cycle, 22–24 °C, 60–70% humidity), at Kunming Medical University, Yunnan, China. Mice were randomized into four groups (*n* = 6/group): (1) MRL/MpJ mice as control group. (2) MRL/lpr mice as LN group. (3) MRL/lpr mice injected with negative control sh-NC. (4) MRL/lpr mice injected with sh-NEAT1 or sh-STING. Mouse-NEAT1 target sequence: GCCTGGTCTACAAAGTGAGTT. Mouse-STING target sequence: CAACATTCGATTCCGAGATAT. Lentiviral particles (2 × 10^8^ TU/mL in 200 μL PBS) were administered via tail vein at 10 and 14 weeks. Mice were euthanized at 18 weeks (pentobarbital sodium, 100 mg/kg) for tissue collection.

### Renal function assessment

Serum creatinine (Scr), blood urea nitrogen (BUN), and 24-h proteinuria were measured in MRL/lpr and control mice at 10, 14, and 18 weeks using commercial kits (Creatinine Assay Kit, Abcam ab65340, Cambridge, UK; BUN Kit, Sigma MAK006, St. Louis, MO).

### Real-time qPCR

Total RNA from PBMCs, HPCs, and tissues was extracted with RNeasy Mini Kit (Qiagen 74104, Hilden, Germany) and reverse transcribed into cDNA using Hifair^®^ II 1st Strand cDNA Synthesis SuperMix for qPCR (Yeasen, Shanghai, China) following the manufacturer’s instruction. The expression of DNMT1, NEAT1, and STING was quantified with a QuantStudio 5 system (Thermo Fisher, Waltham, MA) using SYBR Green qPCR Master Mix (Yeasen, Shanghai, China). GAPDH was used as endogenous controls. The relative expression was analyzed using the 2^−ΔΔCt^ method. The primer sequences are listed as follows: hDNMT1: forward sequence – ATCCCGAGTCCCTGCTG, reverse sequence – TCCTCTGTTGGCTGGGTT; hNEAT1: forward sequence – TTCCCTACTCTTGCCCCTCA, reverse sequence – CATCCTCCACAGGCTTACCG; hSTING: forward sequence – CAGCCTGATGAGCCTTTGGATGAC, reverse sequence – GGACTGGACATGGCACAACTCTTC; hGAPDH: forward sequence – CCAGGTGGTCTCCTCTGA, reverse sequence – GCTGTAGCCAAATCGTTG; mNEAT1: forward sequence – TGGCTAGCTCAGGGCTTCAG, reverse sequence – TCTCCTTGCCAAGCTTCCTTC; mSTING: forward sequence – CCAAGAACCCACAGACGGAAACAG, reverse sequence – GGAGGAGGTGCCACTGAGGTC; mGAPDH: forward sequence – AGCCCAAGATGCCCTTCAGT, reverse sequence – CCGTGTTCCTACCCCCAATG.

### Cell viability assay

HPC cells were plated in 96-well plate at the density of 10,000 cells/well and cultured at 37 °C, 5% CO_2_ for 16 h. The cells were then treated as indicated condition in each figure. After treated with each condition, the cells were treated with 100 μL of CCK-8 solution (CCK-8:serum-free medium = 1:10) and incubated at 37 °C, 5% CO_2_ for 1 h. Podocyte viability was assessed with CCK-8 reagent (PF00004, Proteintech, Rosemont, IL) at 450 nm absorbance.

### Western blot assay

Cells or tissue were collected and lysed using RIPA lysis buffer at 4 °C for 30 min after indicated treatments. The protein concentration was quantified with a BCA Protein Assay Kit (Thermo Fisher Scientific). Proteins (25 μg cell lysate/50 μg tissue) were separated on 10% SDS-PAGE, transferred to PVDF membranes (Millipore ISEQ00010, Burlington, MA). Then, the membranes were blocked with 5% BSA in TPBS for 2 h at room temperature (RT). The membrane was incubated with primary antibodies overnight at 4 °C. The main primary antibodies used are as follows: anti-NLRP3 (Abcam, ab263899, Cambridge, UK, 1:1,000), anti-STING (Proteintech, Rosemont, IL, 19851-1-AP, 1:800), anti-ASC (Santa Cruz, Santa Cruz, CA, sc-514414, 1:500), anti-GSDMD (Proteintech, Rosemont, IL, 66387-Ig, 1:1,000), anti-NLRP3 (Cell Signaling Technology, Danvers, MA, 15,101), anti-β-Actin (Beyotime, Shanghai, China, AF0003, 1:10,000). Then, the membranes were washed three times using TBST buffer and incubated with HRP-conjugated secondary antibodies for 1 h at RT. The binding of primary and secondary antibodies was detected using ECL western blotting analysis system. Relative densities were analyzed using NIH ImageJ software (Bethesda, MD).

### Immunohistochemistry staining

Fresh kidney tissues were fixed in 10% neutral-buffered formalin (NBF) for 24–48 h at 4 °C. Tissues were dehydrated through graded ethanol series (70% → 95% → 100%), cleared in xylene, and embedded in paraffin. Four-micrometer-thick sections were mounted on poly-l-lysine-coated slides. Slides were baked at 60 °C for 30 min, then deparaffinized in xylene (2 × 10 min). Rehydrated through graded ethanol (100% → 95% → 70%; 5 min each) and distilled water. Slides were immersed in 10 mM citrate buffer (pH 6.0) and heated in a pressure cooker (121 °C, 15 psi) for 20 min. Cooled to RT for 30 min. Endogenous peroxidase blocked with 3% H_2_O_2_ in methanol (15 min, RT). Nonspecific sites blocked with 5% BSA in PBS (1 h, RT). Sections incubated with anti-GSDMD antibody (Proteintech, Rosemont, IL, 66387-Ig, 1:200 dilution) overnight at 4 °C. HRP-conjugated goat anti-rabbit IgG (Proteintech, Rosemont, IL, SA00001-2, 1:500 dilution) applied for 1 h at RT. Signal developed with DAB substrate kit (Vector Laboratories, Newark, CA, SK-4100) for 2–5 min. Nuclei counterstained with hematoxylin (Sigma, St. Louis, MO, MHS32) for 1 min. Dehydrated through ethanol/xylene series and mounted with resinous medium (Sigma, St. Louis, MO, 109016).

### Transmission electron microscope

Kidney samples were prepared as described previously. Each group kidney samples were fixed in cacodylate buffer (0.1 M, pH 7.4) containing 2.5% glutaraldehyde and 2.5% paraformaldehyde. Post-fixation, samples were immersed in 1% osmium tetraoxide for 1 h at 4 °C, and then samples were dehydrated using graded alcohol (50, 70, 90, and 100%). Samples were oriented longitudinally and embedded in Epon 812. We sent the samples to Kunming Medical University, Yunnan, China to do transmission electron microscope analysis. Ultrathin sections were cut at 70 nm and contrasted with uranyl acetate and lead citrate, and examined at 80 kV with a transmission electron microscope at various magnifications by a blinded investigator.

### ELISA

Cytokines (IL-6, IL-1β, IL-18, IFN-γ, TNF-α), urine protein (UP) and podocalyxin were detected using ELISA kits according to the manufacturer’s protocol on a BioTek Elx800 reader (Winooski, VT) (Table 1).

**Table 1.** ELISA kit sources and catalog numbers.

Target	Company	Cat #
Human podocalyxin	Elabscience (Wuhan, China)	E-EL-H2360c
Human IL-6	Shanghai Enzyme-linked Biotechnology Co. Ltd. (Shanghai, China)	ml058097
Human IL-1β	Shanghai Enzyme-linked Biotechnology Co. Ltd. (Shanghai, China)	ml058059
Human IL-18	Shanghai Enzyme-linked Biotechnology Co. Ltd. (Shanghai, China)	ml058055
Human TNF-α	Shanghai Enzyme-linked Biotechnology Co. Ltd. (Shanghai, China)	ml077385
Human IFN-γ	Shanghai Enzyme-linked Biotechnology Co. Ltd. (Shanghai, China)	ml077386
Mouse podocalyxin	Elabscience (Wuhan, China)	E-EL-H2360c
Mouse IL-6	Shanghai Enzyme-linked Biotechnology Co. Ltd. (Shanghai, China)	ml063159
Mouse IL-1β	Shanghai Enzyme-linked Biotechnology Co. Ltd. (Shanghai, China)	ml098416
Mouse IL-18	Shanghai Enzyme-linked Biotechnology Co. Ltd. (Shanghai, China)	Ml002294V
Mouse TNF-α	Shanghai Enzyme-linked Biotechnology Co. Ltd. (Shanghai, China)	ml002095
Mouse IFN-γ	Shanghai TW-reagent (Shanghai, China)	TW8439
Mouse urine protein	Shanghai Enzyme-linked Biotechnology Co. Ltd. (Shanghai, China)	MM-44287M2

### RNA immunoprecipitation assay

RNA immunoprecipitation (RIP) assay was performed according to manufacturer’s protocol (Millipore Corporation, Burlington, MA). Briefly, cells were harvested and lysed followed by incubation with the detection antibody at 4 °C overnight with gentle rotation. The protein A/G beads (40 μL) were added and incubated with lysate for 1 h at 4 °C to capture the complex. After washing in the buffer, RNA was isolated for detection of its concentration by RT-qPCR.

### RNA pull-down assay

Fifty microliters magnetic beads were washed three times with 1 mL wash buffer, then biotin-labeled NEAT1 probe (Shanghai Bioengineering Co., Inc., Shanghai, China) was added and incubated at 4 °C for 2 h. The sample was placed on a magnetic mount to remove the supernatant, and the prepared cell lysate was added and incubated at 4 °C overnight to precipitate the immune complex of RNA binding proteins. After washing three times with Wash buffer, the protein samples were eluted and DNMT1 protein was detected by Western blot.

### Statistical analysis

Data are expressed as mean ± SEM (*n* ≥ 5). Normality was verified via Shapiro–Wilk tests. Comparisons used one-way ANOVA with Bonferroni’s correction or Welch’s ANOVA for heteroscedastic data (Prism 8.0, GraphPad, La Jolla, CA). Significance threshold: *p < 0.05.

## Results

### NEAT1 mediates LN-IgG-induced inflammatory cytokine release in podocytes

**Figure 1.**
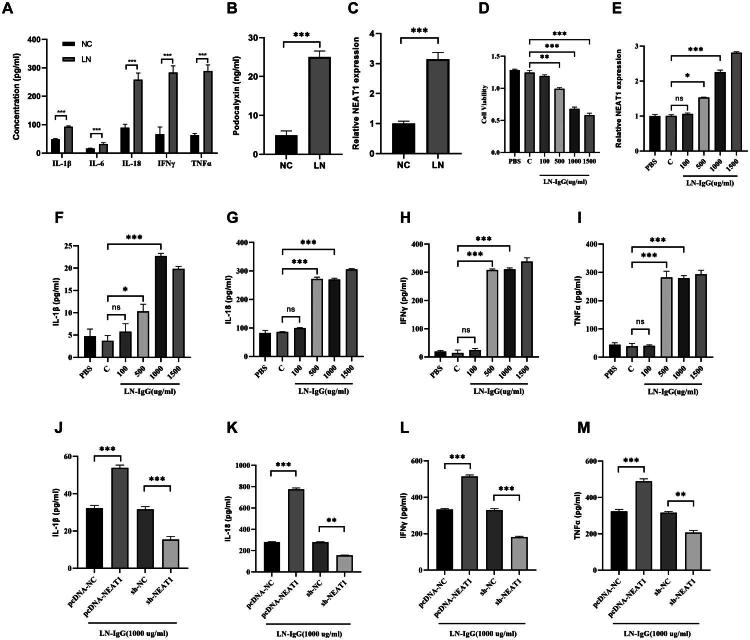
LN-IgG stimulates podocyte injury and inflammatory cytokine expression through NEAT1. (A) ELISA quantification of plasma inflammatory cytokines (IL-1β, IL-6, IL-18, IFN-γ, TNF-α) in the lupus nephritis (LN) patients and healthy controls (NC). (B) ELISA quantification of urinary podocalyxin in LN patients and NC. (C) RT-qPCR analysis of NEAT1 expression in PBMCs from LN patients and NC. (D) CCK-8 assay showing viability of HPCs treated with increasing concentrations of LN-IgG (100, 500, 1,000, and 1,500 μg/mL) or control IgG (C, 1,500 μg/mL) for 48 h. (E) RT-qPCR analysis of NEAT1 expression in HPCs treated as in (D). (F–I) ELISA quantification of inflammatory cytokines (IL-1β, IL-18, IFN-γ, TNF-α) in supernatants of HPCs from (D). (J–M) ELISA analysis of cytokine levels in HPCs transfected with pcDNA-NC, pcDNA-NEAT1, sh-NC, or sh-NEAT1, followed by treatment with 1,000 μg/mL LN-IgG for 48 h. Data are presented as mean ± SD. ns: not significant; *p < 0.05, **p < 0.01, and ***p < 0.001.

SLE patients exhibit elevated circulating inflammatory cytokines [20,21]. LN representing a severe renal complication. To characterize the inflammatory profile of LN, we quantified plasma cytokines in 25 treatment-naïve LN patients and 20 healthy donors (NC). ELISA analysis revealed significant increases in IL-1β, IL-6, IL-18, IFN-γ, and TNF-α compared to NC (*p* < 0.001, Figure 1(A)). Notably, urinary podocalyxin – a biomarker of podocyte injury – was elevated fivefold in LN patients (*p* < 0.001, Figure 1(B)), correlating with disease activity (SLEDAI-2000) [22]. Mechanistically, we identified NEAT1 as a potential mediator of podocyte dysfunction. PBMCs from LN patients exhibited threefold higher NEAT1 expression than NC (*p* < 0.001, Figure 1(C)), consistent with prior reports of NEAT1 upregulation in LPS-stimulated human renal mesangial cells (HRMCs). To establish causality, we isolated IgG from LN patient plasma and treated HPCs *in vitro*. LN-IgG induced dose-dependent cytotoxicity, reducing cell viability to 61 ± 7% at 1,000 μg/mL (vs. NC, *p* < 0.001, Figure 1(D)), paralleled by NEAT1 upregulation (2.3-fold at 1,000 μg/mL, *p* < 0.001, Figure 1(E)) and increased secretion of IL-1β, IL-18, IFN-γ, and TNF-α (Figure 1(F–I)). NC-IgG (1,500 μg/mL) showed no cytotoxicity or cytokine induction (*p* > 0.05), confirming LN-IgG specificity. Gain- and loss-of-function studies demonstrated NEAT1’s central role in this process. Overexpression of NEAT1 (pcDNA-NEAT1) exacerbated LN-IgG-induced inflammatory cytokines release (vs. pcDNA-NC, *p* < 0.001), whereas NEAT1 knockdown (sh-NEAT1) attenuated cytokine production (vs. sh-NC, *p* < 0.001) (Figure 1(J–M)). These findings imply that NEAT1 maybe as a critical amplifier of LN-IgG-driven podocyte injury and inflammation.

### NEAT1 drives podocyte injury and renal inflammation in murine lupus nephritis

**Figure 2.**
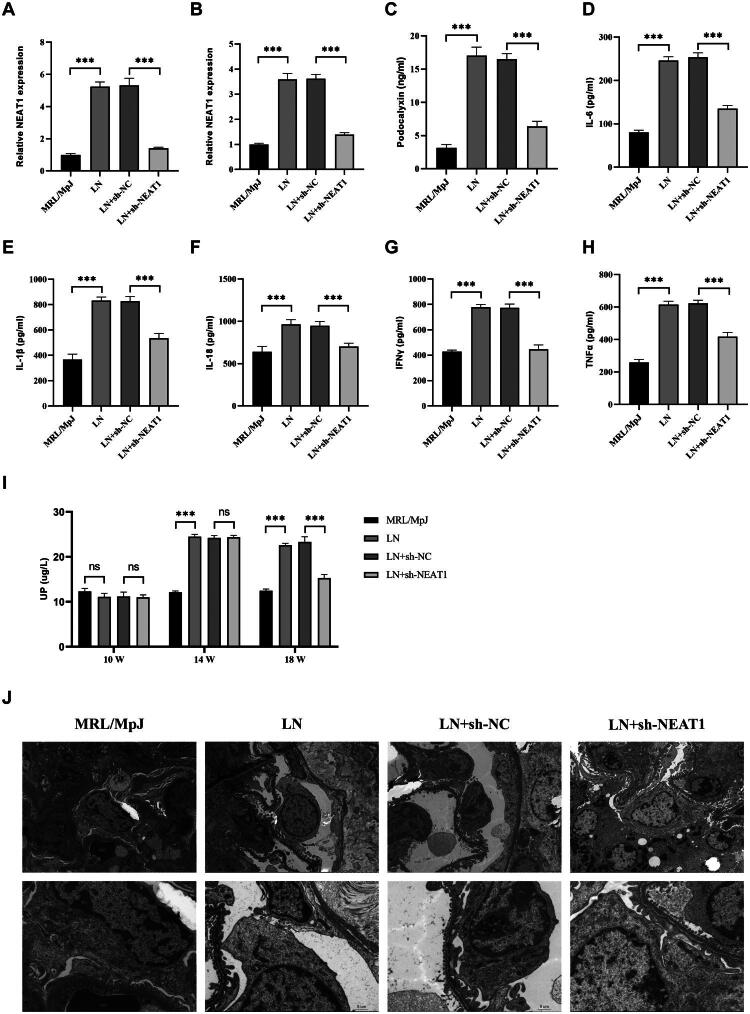
NEAT1 promotes podocyte injury and inflammatory cytokine release in LN mice. (A) RT-qPCR analysis of NEAT1 expression in PBMCs from MRL/MpJ control mice, MRL/lpr LN mice, and LN mice injected with sh-NC or sh-NEAT1 lentivirus. (B) RT-qPCR analysis of renal NEAT1 expression in the indicated groups. (C) ELISA quantification of urinary podocalyxin. (D–H) ELISA quantification of inflammatory cytokines (IL-6, IL-1β, IL-18, IFN-γ, TNF-α) in the plasma of the indicated groups. Data are presented as mean ± SD. (I) ELISA quantification of urine protein. (J) Electron microscope image of mouse kidney. ns: not significant; ***p < 0.001.

To establish the *in vivo* relevance of NEAT1 in LN pathogenesis, we employed the MRL/lpr lupus-prone mouse model. Compared to MRL/MpJ control mice, MRL/lpr mice exhibited fivefold higher NEAT1 expression in PBMCs (*p* < 0.001, Figure 2(A)) and threefold elevation in renal tissues (*p* < 0.001, Figure 2(B)) at 18 weeks of age. This upregulation correlated with progressive podocyte injury, as evidenced by fourfold higher urinary podocalyxin excretion (*p* < 0.001, Figure 2(C)) and twofold higher UP excretion (*p* < 0.001, Figure 2(I)). Lentiviral delivery of sh-NEAT1 via tail vein injection achieved renal NEAT1 knockdown, which significantly attenuated urinary podocalyxin levels (*p* < 0.001, Figure 2(C)). Systemic inflammation was concomitantly reduced, with decreased plasma IL-1β, IL-6, IL-18, IFN-γ, and TNF-α (*p* < 0.001, Figure 2(D–H)). The electron microscope results of the mouse kidneys showed that in the LN group, the podocytes (SC) exhibited pyroptosis-like changes, possessing the characteristics of pyroptotic cells. The cell membrane (→) was locally damaged, and multivesicular bodies (Mvb) were present. In the sh-NEAT1 group, the podocytes showed no edema, the cytoplasm was uniform, and the cell membrane structure was intact (Figure 2(J)).

### LN-IgG induced podocytes pyroptosis through NEAT1

**Figure 3.**
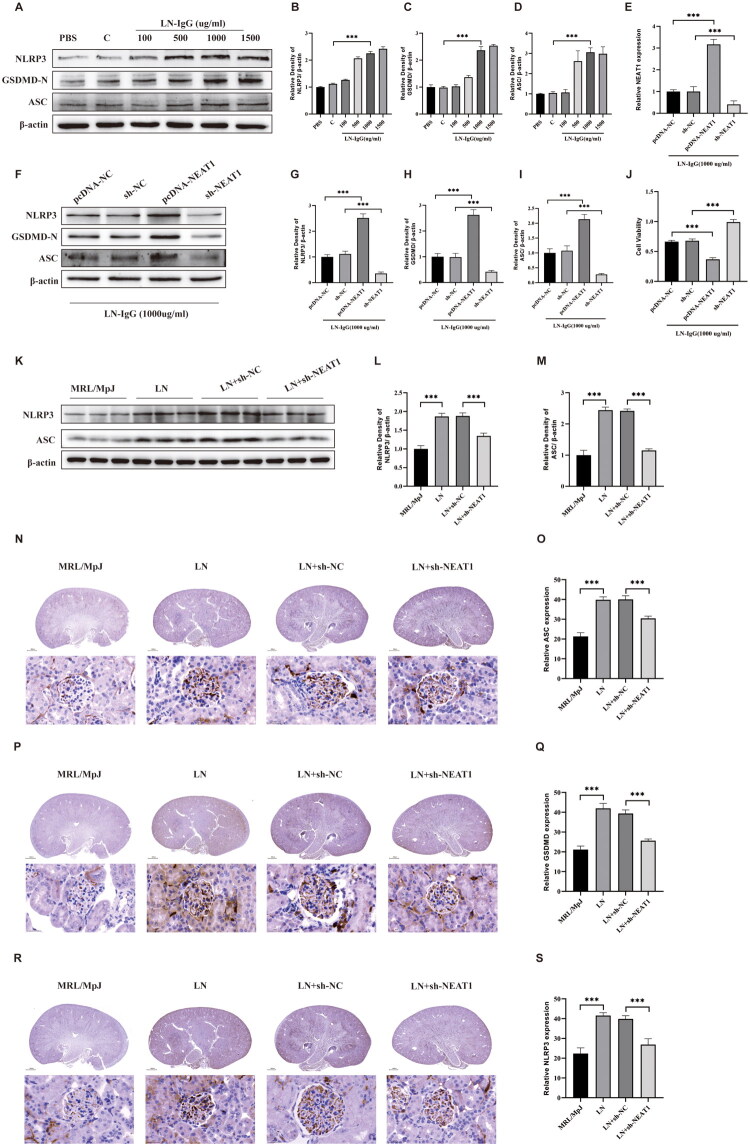
LN-IgG induces podocyte pyroptosis via NEAT1. (A–D) Western blot analysis of pyroptosis-related proteins (NLRP3, ASC, GSDMD-N) in HPCs treated with LN-IgG (100–1,500 μg/mL). (E) RT-qPCR analysis of NEAT1 expression in HPCs transfected with pcDNA-NC, pcDNA-NEAT1, sh-NC, or sh-NEAT1, followed by 1,000 μg/mL LN-IgG treatment. (F–I) Western blot analysis of NLRP3, ASC, and GSDMD-N under corresponding treatments. (J) CCK-8 assay of cell viability under corresponding treatments. (K–M) Western blot analysis of renal NLRP3 and ASC expression in the indicated group. (N, O) Immunohistochemical staining and analysis of ASC in mouse kidney sections (scale bar: 25 μm). (P, Q) Immunohistochemical staining and analysis of GSDMD in mouse kidney sections (scale bar: 25 μm). (R, S) Immunohistochemical staining and analysis of NLRP3 in mouse kidney sections (scale bar: 25 μm). Western blot band intensities were quantified using ImageJ, with β-actin as loading control. Data are presented as mean ± SD. ns: not significant; ***p < 0.001.

The elevated IL-1β/IL-18 secretion (Figure 1(F,G,J,K)) suggested LN-IgG might trigger pyroptosis – a lytic cell death mechanism characterized by gasdermin pore formation. NLRP3 inflammasomes play key roles in cell pyroptosis [23]. Immunoblotting revealed that LN-IgG (100–1,500 μg/mL) dose-dependently upregulated pyroptosis executors in HPCs: NLRP3, ASC, and cleaved GSDMD (GSDMD-N) (vs. control, *p* < 0.001, Figure 3(A)). Given prior reports of NEAT1 regulating NLRP3 in macrophages, we interrogated its role in podocytes [18]. NEAT1 overexpression (pcDNA-NEAT1) synergized with LN-IgG to amplify NLRP3, ASC, and GSDMD-N (Figure 3(E–I)). Conversely, NEAT1 knockdown (sh-NEAT1) suppressed these effects (vs. sh-NC, Figure 3(E–I)). Functionally, NEAT1 overexpression exacerbated cell death (vs. pcDNA-NC, *p* < 0.001), while NEAT1 knockdown improved survival (Figure 3(J)). *In vivo* validation showed MRL/lpr mice exhibited higher renal NLRP3 (Figure 3(K,L,R,S)), ASC (Figure 3(K,M,N,O)), and GSDMD^+^ (Figure 3(P,Q)) cells (*p* < 0.001) than MRL/MpJ controls. Lentiviral sh-NEAT1 reduced NLRP3, ASC, and GSDMD^+^ cells (*p* < 0.001), confirming NEAT1’s conserved role across species.

### NEAT1 potentiates podocyte pyroptosis via STING upregulation

**Figure 4.**
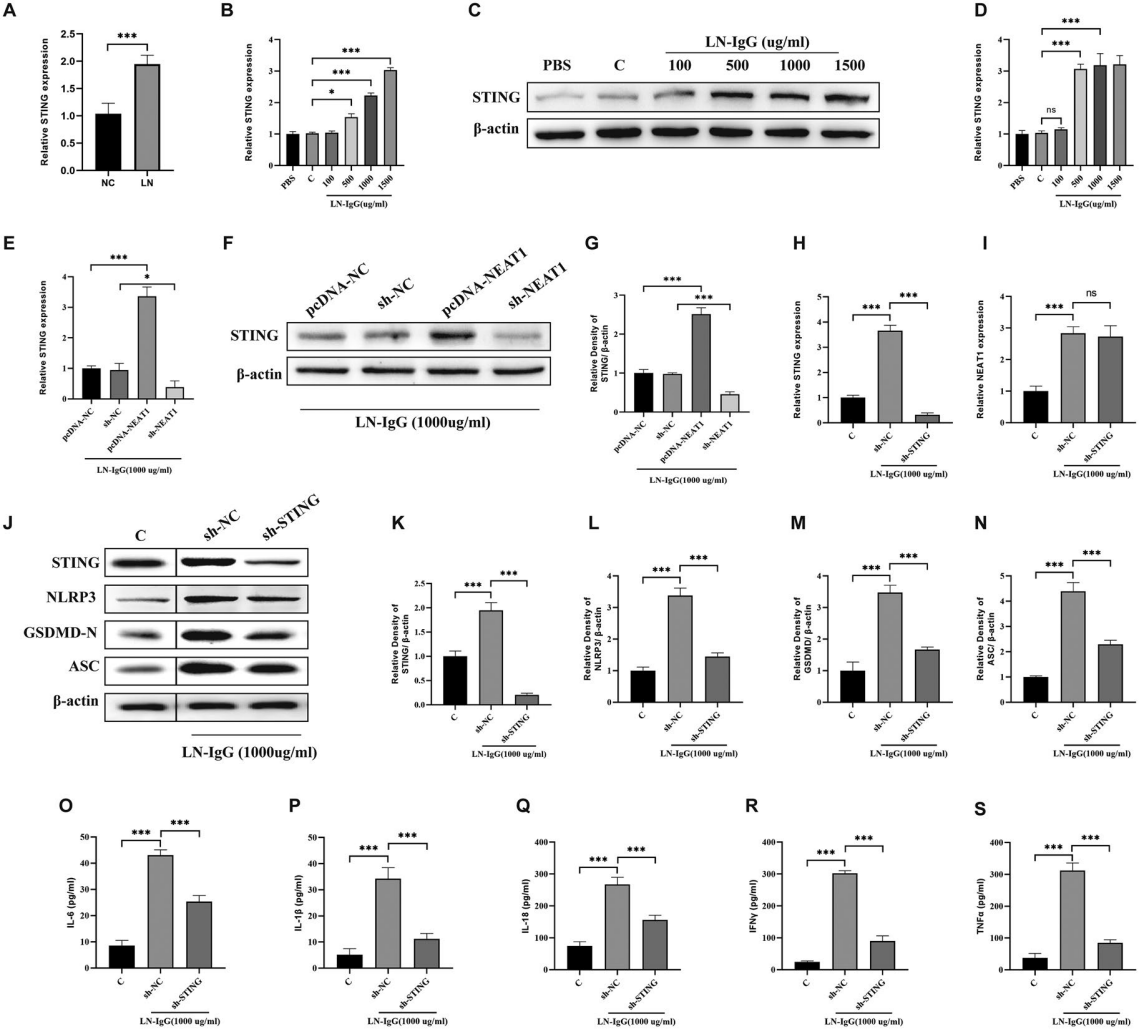
NEAT1 mediates inflammatory cytokine secretion through STING. (A) RT-qPCR analysis of STING expression in PBMCs from LN patients and NC. (B–D) RT-qPCR and Western blot analysis of STING expression in HPCs treated with LN-IgG (500–1,500 μg/mL). (E, F) STING mRNA and protein levels in HPCs from Figure 1(D) treatment groups. (G–I) RT-qPCR analysis of STING and NEAT1 expression in HPCs respectively transfected with sh-NC, sh-NEAT1 or sh-STING, followed by 1,000 μg/mL LN-IgG treatment. (J–N) Western blot analysis of NLRP3, GSDMD-N, and ASC in corresponding groups. (O–S) ELISA quantification of inflammatory cytokines (IL-6, IL-1β, IL-18, IFN-γ, TNF-α) in supernatants with corresponding groups. Western blot band intensities were quantified using ImageJ, with β-actin as loading control. Data are presented as mean ± SD. ns: not significant; *p < 0.05, ***p < 0.001.

STING has been reported to play critical role in SLE and LN disease phenotypes by using mouse model [24,25]. In this study, we aimed to elucidate whether STING plays a role in the NEAT1 mediated podocytes pyroptosis. We demonstrated that NEAT1 expression level is elevated in LN patient PBMCs. Consistent with this notion, STING mRNA was elevated 2.8-fold in LN patient PBMCs (*p* < 0.001, Figure 4(A)) and showed strong positive correlation with NEAT1 levels. *In vitro*, LN-IgG (1,000 μg/mL) concomitantly upregulated NEAT1 and STING in podocytes (vs. control, *p* < 0.001, Figures 1(E) and 4(B–E)). These data indicated that STING expression is positively correlated with NEAT1 expression. NEAT1 over expression increased STING expression in HPCs, and knockdown NEAT1 expression reduced STING expression (Figure 4(F,G)), which suggested that NEAT1 bidirectionally regulated STING. Notably, we found that knockdown of STING expression using sh-STING in HPCs did not affect the expression level of NEAT1, implying that STING may play a role downstream of NEAT1 (Figure 4(H,I)). Functional studies demonstrated that STING knockdown: abrogated LN-IgG-induced pyroptosis markers (NLRP3, ASC, and GSDMD-N) (Figure 4(J–N)); attenuated cytokine release (IL-6, IL-1β, IL-18, IFN-γ, and TNF-α) (Figure 4(O–S)). These data position STING as the critical executor of NEAT1-driven pyroptosis, independent of feedback regulation.

### STING knockdown attenuates renal pyroptosis in lupus nephritis mice

**Figure 5.**
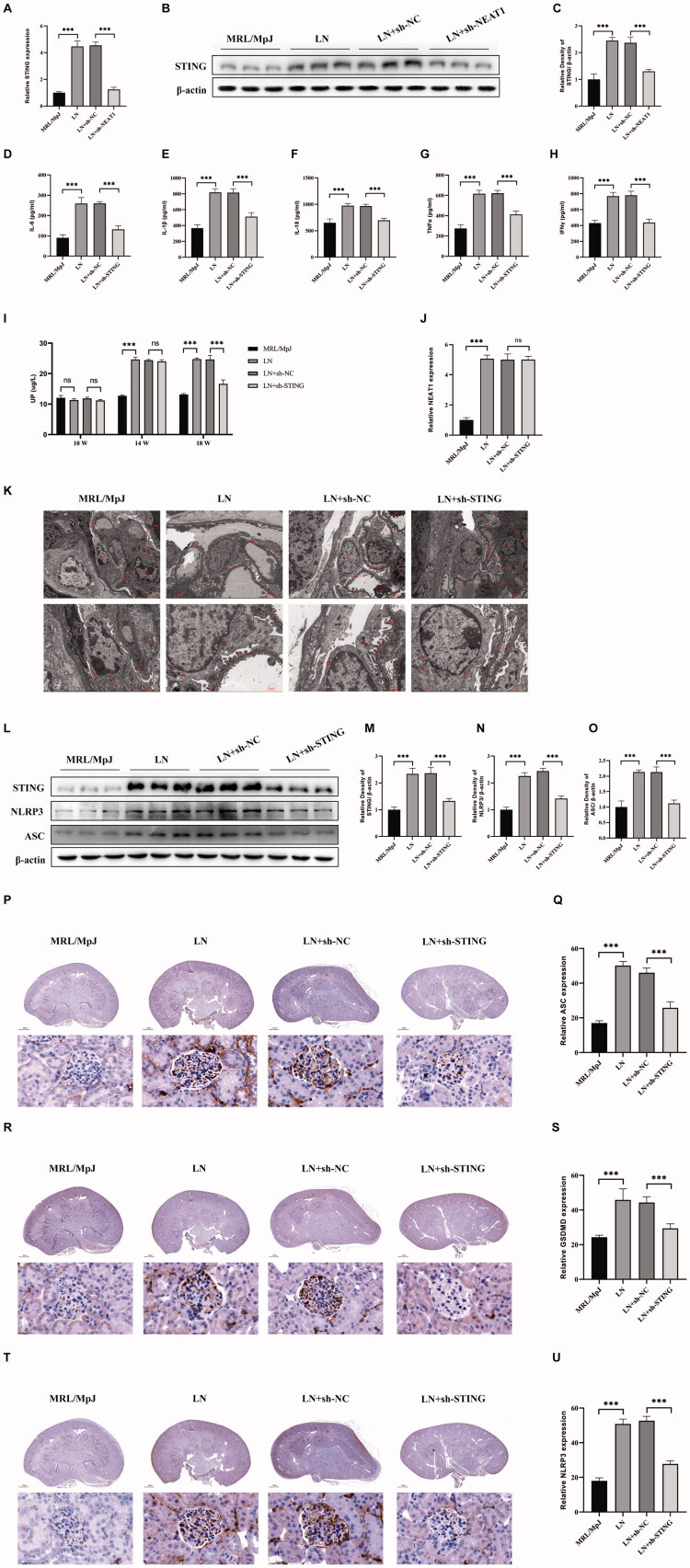
STING knockdown attenuates podocyte pyroptosis in LN mice. (A–C) RT-qPCR and Western blot analysis of renal STING expression in MRL/MpJ control, MRL/lpr LN, and LN mice injected with sh-NC or sh-NEAT1. (D–H) ELISA quantification of inflammatory cytokines (IL-6, IL-1β, IL-18, INF-γ, TNF-α) in MRL/MpJ control, MRL/lpr LN, and LN mice injected with sh-NC or sh-STING. (I) ELISA quantification of urine protein. (J) RT-qPCR analysis of renal NEAT1 expression in corresponding groups. (K) Electron microscope image of mouse kidney. (L–O) Western blot analysis of renal STING, NLRP3, and ASC expression in corresponding groups. (P, Q) Immunohistochemical staining and analysis of ASC in mouse kidney sections (scale bar: 25 μm). (R, S) Immunohistochemical staining and analysis of GSDMD in mouse kidney sections (scale bar: 25 μm). (T, U) Immunohistochemical staining and analysis of NLRP3 in mouse kidney sections (scale bar: 25 μm). Western blot band intensities were quantified using Image J, with β-actin as loading control. Data are presented as mean ± SD. ns: not significant; ***p < 0.001.

To validate the NEAT1/STING axis *in vivo*, we assessed STING expression in renal tissues of MRL/lpr lupus-prone mice. Compared to MRL/MpJ controls, LN mice exhibited higher STING mRNA (*p* < 0.001) and elevated STING protein (*p* < 0.001) levels, consistent with prior reports of STING upregulation in autoimmune renal injury (Figure 5(A–C)). Lentiviral sh-NEAT1 delivery reduced renal STING expression (vs. sh-NC, *p* < 0.001, Figure 5(A–C)), confirming NEAT1-dependent STING regulation. To dissect STING’s functional role, we administered sh-STING via tail vein injection. Successful STING knockdown (*p* < 0.001, Figure 5(L,M)) attenuated systemic inflammation, reducing plasma inflammatory cytokines (*p* < 0.001, Figure 5(D–H)) and UP excretion (*p* < 0.001, Figure 5(I)). The electron microscopy results of mouse kidneys showed that the knockdown of STING had the same outcome as the knockdown of NEAT1: the pyroptosis morphology of podocytes was improved (Figure 5(K)). Crucially, STING silencing suppressed pyroptosis markers: NLRP3 (*p* < 0.001, Figure 5(L,N,T,U)), ASC (*p* < 0.001, Figure 5(L,O–Q)), and GSDMD-N (*p* < 0.001, Figure 5(R,S)), aligning with studies showing NLRP3 inflammasome suppression mitigates pyroptosis in renal pathologies. Notably, STING knockdown did not alter NEAT1 expression (Figure 5(J)), confirming STING acts strictly downstream of NEAT1. This unidirectional relationship is supported by recent findings that lncRNAs regulate STING signaling without feedback loops [26,27].

### NEAT1 may recruits DNMT1 to epigenetically regulate STING in LN

**Figure 6.**
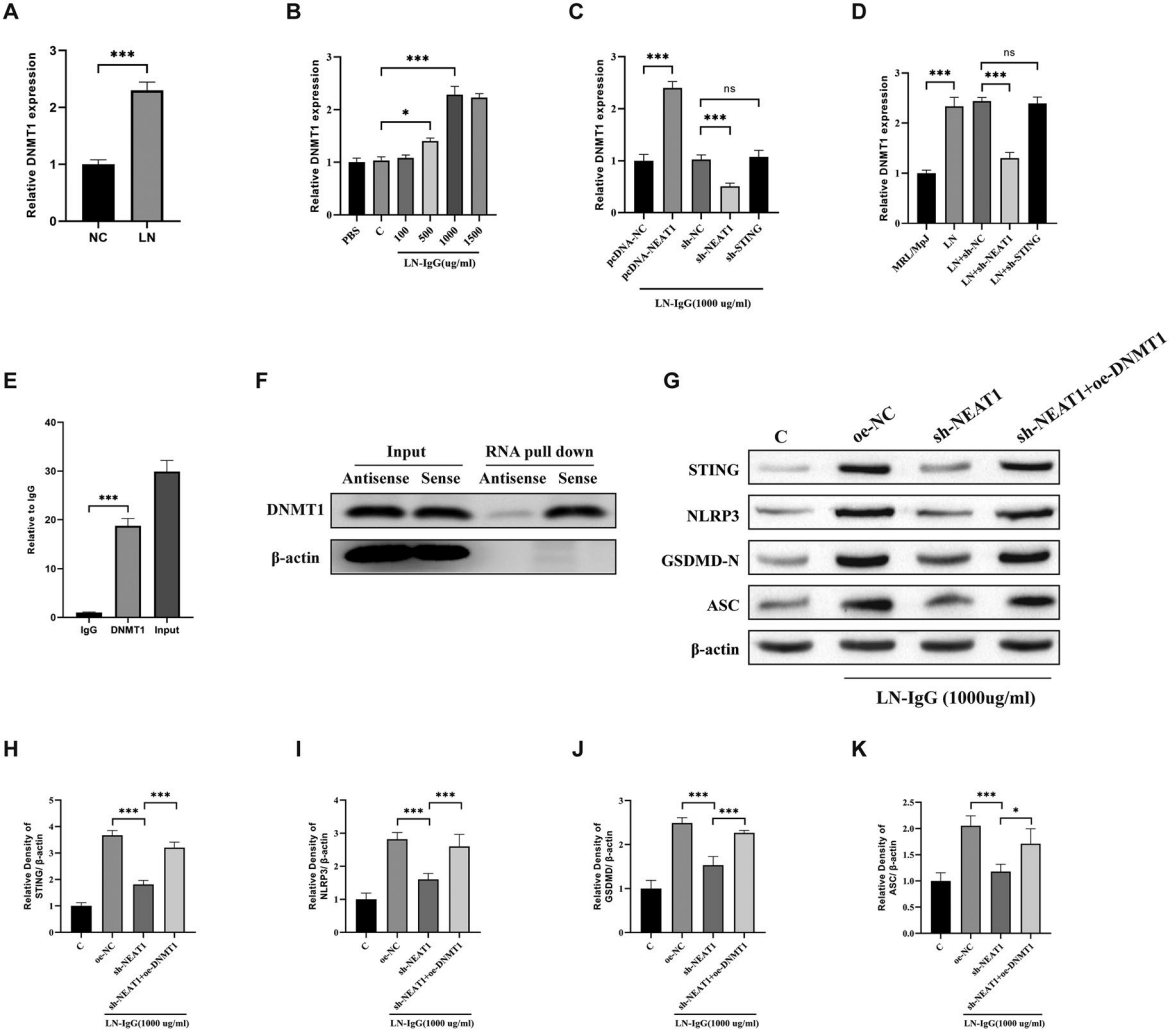
NEAT1 interacts with DNMT1 and regulates its expression in LN. (A) RT-qPCR analysis of DNMT1 expression in PBMCs from LN patients and NC. (B) DNMT1 mRNA levels in HPCs treated with LN-IgG (100–1,500 μg/mL). (C) DNMT1 expression in HPCs transfected with the indicated plasmids, followed by LN-IgG treatment. (D) RT-qPCR analysis of renal DNMT1 expression in mouse groups. (E) RIP-qPCR showing enrichment of NEAT1 in DNMT1 immunoprecipitates (vs. IgG control). (F) RNA pull-down assay confirming DNMT1 binding to biotinylated NEAT1 probes. (G–K) Western blot analysis of STING, NLRP3, GSDMD-N, and ASC under corresponding treatments. Data are presented as mean ± SD. ns: not significant; *p < 0.05 and ***p < 0.001.

DNA methyltransferase 1, a key enzyme mediating CpG island methylation, has been implicated in lupus pathogenesis through aberrant epigenetic regulation [28,29]. Building on reports that NEAT1 interacts with DNMT1 in cancer models, we investigated this axis in LN [30,31]. Clinical analysis revealed 2.2-fold higher DNMT1 mRNA in LN patient PBMCs compared to healthy donors (*p* < 0.001, Figure 6(A)), paralleling NEAT1 upregulation. *In vitro*, LN-IgG (1,000 μg/mL) induced dose-dependent DNMT1 upregulation (2.3-fold vs. control, *p* < 0.001, Figure 6(B)), which was abolished by NEAT1 knockdown (Figure 6(C)). Notably, STING silencing did not affect DNMT1 expression (Figure 6(C)). Instead, overexpression of DNMT1 increased the expression of STING and eliminated the effect of sh-NEAT1 on the cell pyroptosis marker: NLRP3, GSDMD, and ASC (*p* < 0.001, Figure 6(G–K)), confirming DNMT1 operates upstream of STING. Mechanistically, RIP assays showed 18-fold enrichment of NEAT1 in DNMT1 immunoprecipitates (vs. IgG control, *p* < 0.001, Figure 6(E)). RNA pull-down further confirmed direct NEAT1–DNMT1 binding (Figure 6(F)), consistent with findings that lncRNAs scaffold DNA methyltransferases to specific genomic loci. *In vivo*, MRL/lpr mice exhibited 2.3-fold higher renal DNMT1 (*p* < 0.001), which was normalized by NEAT1 knockdown (vs. LN + sh-NC, *p* < 0.001, Figure 6(D)). These data establish a NEAT1/DNMT1/STING regulatory axis, where NEAT1 recruits DNMT1 to modulate STING methylation – a mechanism recently implicated in autoimmune gene silencing.

## Discussion

LN is one of serious complication of SLE. Despite advances in LN treatment, most of the current treatments are immunosuppressive drugs, which only relief symptoms [9]. The pathogenesis of LN is complicated and not currently completely understood. Given the high percentage of SLE patients affected by LN, finding novel effective treatments via discovering the possible pathogenesis of LN is significantly needed.

Podocytes as the key player of the glomerular filtration barrier in the kidney, when injured or dying lead to the proteinuria, which is a hallmark of LN [9]. LN-IgG can attack, damage podocytes. In turn, podocytes can respond to the damage and secret inflammatory cytokines, causing further damage to the kidney. Therefore, protecting podocytes from damage and preventing inflammatory cytokines release could be a therapeutic target. Our data presented here confirmed that LN-IgG damaged podocytes as indicated by podocalyxin release, and showed that LN-IgG increased secretion of inflammatory cytokines from podocytes (Figure 1). Long non-coding RNA NEAT1 was identified as a cancer biomarker and has been found to play significant roles in a few various cancers [32,33]. NEAT1 was also found to play significant roles in diabetic nephropathy in a rat model [19]. In this study, we proved that HPCs responded to LN-IgG by increasing NEAT1 expression (Figure 1), which positively correlated with increased inflammatory cytokines release. Cell pyroptosis, in both canonical and non-canonical pathways, result in release of IL-1β and IL-18 [34]. Gain and loss-of-function studies indicated that NEAT1 may play a role in podocytes pyroptosis, as indicated by release of IL-1β and IL-18, and by protein expression level of NLRP3, GSDMD, and ASC.

Stimulator of interferon genes is cyclic dinucleotides sensor and a key player of innate immune response [35,36]. STING knockout mice are susceptible to lethal infection but are general healthy when kept in pathogen free environment [37]. Given that STING plays role in podocytes inflammation and damage, it could be an excellent therapeutic target for LN [25,38,39]. There is no evidence showed that STING directly or indirectly interacts with NEAT1. We found that NEAT1 induced STING expression in podocytes, and NEAT1 knockdown reduced STING expression. However, knockdown of STING had no effect on the expression level of NEAT1. This study showed that STING may play a role downstream of NEAT1 and activating NLRP3 inflammasome and inducing pyroptosis in podocytes. We are further investigating the molecular mechanisms of NEAT1 regulating STING1 gene. The endogenous binding of expression, DNMT1 and NEAT1 provides a direction of investigation that we are interested in. The results will be published later.

In summary, our data demonstrated that podocytes responded to LN-IgG, resulting in podocyte damage, inflammatory cytokines release, and pyroptosis, which were at least in part mediated by NEAT1. Moreover, STING may play a role downstream of NEAT1 in the podocyte damage, inflammatory cytokines release, and pyroptosis. However, the detailed molecular mechanisms warrant further study. In conclusion, NEAT1/DNMT1/STING signaling axis could be therapeutic targets for LN.

## Supplementary Material

Supplemental Material

## Data Availability

All data generated or analyzed during this study are included in this published article.
