# Supplementary material for: Long non-coding RNA NEAT1 mediated lupus nephritis induced podocytes pyroptosis through DNMT1–STING axis
Source: Ren Fail. 2025 Dec 28;47(1):2595372. doi: 10.1080/0886022X.2025.2595372 (PMC12777783; doi:10.1080/0886022X.2025.2595372)
Supplement: Supplemental Material [file IRNF_A_2595372_SM4057.doc]

**Table 1**. The clinical characteristic of patients with LN

| Patient | Age | Gender | SLEDAI(score) | eGFR(ml/min/1.73m²) | URTP(g/day) | Class |
| --- | --- | --- | --- | --- | --- | --- |
| 1 | 35 | F | 14 | 96 | 1.8 | III |
| 2 | 22 | F | 25 | 121 | 5.6 | III+V |
| 3 | 38 | F | 18 | 128.2 | 8.2 | V |
| 4 | 41 | F | 20 | 85.3 | 4.1 | IV+V |
| 5 | 19 | M | 15 | 129.4 | 0.6 | II |
| 6 | 26 | M | 21 | 120.5 | 5.7 | III+V |
| 7 | 52 | F | 15 | 76.8 | 1.2 | III |
| 8 | 47 | F | 17 | 82.3 | 1.6 | IV |
| 9 | 24 | M | 21 | 56.1 | 3.7 | IV |
| 10 | 28 | F | 23 | 72.2 | 4.3 | III+V |
| 11 | 38 | F | 19 | 82.5 | 0.8 | II |
| 12 | 34 | F | 20 | 98.4 | 1.7 | V |
| 13 | 42 | F | 16 | 77.9 | 3.9 | IV+V |
| 14 | 49 | M | 23 | 69.1 | 0.9 | III |
| 15 | 51 | F | 15 | 52.5 | 3.2 | IV+V |
| 16 | 35 | F | 16 | 98.2 | 1.7 | V |
| 17 | 29 | F | 24 | 112.3 | 6.2 | IV+V |
| 18 | 36 | F | 18 | 65.3 | 4.9 | V |
| 19 | 58 | F | 17 | 48.2 | 3.4 | III+V |
| 20 | 17 | F | 22 | 124.1 | 7.2 | V |
| 21 | 28 | M | 19 | 78.3 | 1.4 | III |
| 22 | 34 | F | 20 | 95.2 | 5.5 | IV |
| 23 | 59 | M | 21 | 46.1 | 1.4 | IV |
| 24 | 32 | F | 22 | 96.5 | 4.3 | IV+V |
| 25 | 46 | F | 16 | 65.2 | 2.4 | III |

SLEDAI systemic lupus erythematosus disease activity index; eGFR estimated glomerular filtration rate based on CKD-EPI formula; URTP urinary total proteinuria.
